# Delaying aging by improved hydration

**DOI:** 10.1016/j.ebiom.2023.104638

**Published:** 2023-05-26

**Authors:** Natalia I. Dmitrieva, Douglas R. Rosing, Manfred Boehm

**Affiliations:** a Laboratory of Cardiovascular Regenerative Medicine, Translational Vascular Medicine Branch, NHLBI, Bethesda, MD, USA; b Cardiovascular Branch Clinical, NHLBI, Bethesda, MD, USA

We would like to thank Dr. Ortiz for suggesting that chronic kidney disease (CKD) rather than hypohydration per se may underlie the increased risks of negative long-term health outcomes. We agree that it is a valid hypothesis for testing in future studies, including interventional trials that are needed for reaching definite conclusions. However, we believe that available information can already be put into practice. Thus, 142 mmol/l serum sodium threshold can be safely incorporated into clinical practice and everyday life. Based on this threshold, we recently proposed a strategy for making decisions about more comprehensive clinical evaluations, treatments, and fluid intake recommendations.^1^ Increasing water intake to the generally recommended levels is an integral part of the strategy. Applying this strategy would also facilitate early CKD diagnosis and initiation of treatment as Dr. Ortiz suggests. Concerns about dangers of fatal water intoxication are often overestimated. The maximal excretion rate of even minimally diseased kidneys is 0.8–1 L per hour, meaning that it can excrete up to 24 L of water a day^2^ that by far exceeds general recommendations. To prevent water intoxication, the rate of water gain should not exceed the maximal excretion rate that can be ensured through educational information.^2^^,^^3^ Most cases of hyponatremia caused by excessive drinking occur when a large amount of water is consumed over a short period of time.^3^ Variability of individual water needs^4^ does not negate the importance of optimal hydration, but rather calls for the development of better methodology that would allow for the derivation of personalized fluid intake recommendations depending on environmental, demographic and lifestyle factors. Such methods could be useful, for example, to address the emerging epidemic of CKD of unknown origin among young agricultural and construction workers in hot, humid parts of the world. Exposure to heat and persistent dehydration is suspected to be the main causing factor.^5^ To summarize, although additional evidence is needed, why not try to improve hydration for the potential risk-free chance of longer disease-free life?

## Contributors

All authors contributed to conceptualization and writing, read and approved the final version of the response letter.

## Declaration of interests

The authors declare that there is no conflict of interest.
